# How is wakeful rest operationalized and measured in daily life among adults with and without long‐term conditions? A systematic scoping review

**DOI:** 10.1002/pmrj.70008

**Published:** 2025-09-04

**Authors:** Martin Ackah, Katie L. Hackett, Vincent Deary, Florentina Johanna Hettinga, Hosea Boakye, Ulric Sena Abonie

**Affiliations:** ^1^ Department of Sport Exercise and Rehabilitation Northumbria University Newcastle upon Tyne UK; ^2^ Department of Social Work, Education and Community Wellbeing Northumbria University Newcastle upon Tyne UK; ^3^ Department of Psychology Northumbria University Newcastle upon Tyne UK; ^4^ Department of Human Movement Sciences Vrije Universiteit Amsterdam Amsterdam The Netherlands; ^5^ Department of Rehabilitation Sciences Boston University Boston Massachusetts USA

## Abstract

A systematic scoping review was conducted to examine how rest is operationalized and measured in daily life among adults with and without long‐term conditions. Searches were performed in PubMed, Cumulative Index to Nursing and Allied Health Literature, and Psych databases for studies from 1990 to 2024. Two independent reviewers screened and selected eligible studies, which included adults with and without long‐term conditions. A content analysis was used to synthesize qualitative and quantitative evidence, categorizing rest‐related descriptions. Additionally, descriptive and narrative synthesis methods were employed. Of 9393 initial records, 17 studies were included in the review. The findings revealed that rest was operationalized by several key elements, including cessation of activity or engagement in low‐energy tasks (*n* = 9 studies); detachment from activities (*n* = 4 studies); experiences of peace, joy, and tranquility (*n* = 5 studies); and time for self‐reflection and solitude (*n* = 2 studies). The operationalization of rest showed both similarities and differences between adults with and without long‐term conditions. Although all groups defined rest in physical, emotional, and social terms, mental rest was more prominently emphasized by adults without long‐term conditions. The review also identified the effects of rest, including improvements in well‐being and psychological functioning (*n* = 4 studies) and enhanced energy levels that facilitated daily activities (*n* = 4 studies). However, “excessive” rest was linked to negative outcomes, such as increased physical symptoms and disability (*n* = 3 studies). Notably, only two studies assessed rest measurement tools for adults with long‐term conditions. The findings suggest that rest can enhance energy, well‐being, and functioning, but excessive rest may worsen physical symptoms and disability. Tailored guidance on optimal rest is essential for maximizing health benefits. The review also highlights the need for further research on comprehensive tools to measure rest.

## INTRODUCTION

Wakeful rest, hereafter referred to as ‘rest,’ is a state in which an individual remains awake but is not actively engaged in demanding primary activities, thereby supporting optimal daily performance and self‐care.^1^, ^2^, ^3^, ^4^, ^5^ Our recent review revealed that rest was inadequately reported within rehabilitation interventions.^6^ When described, the most reported forms of rest included physical rest, active rest, and engaging in relaxation activities.^6^ Additionally, Bernhofer conceptualizes rest as a multidimensional construct comprising physical, mental, and spiritual components.^7^ In contemporary society, where constant activity is often prioritized, rest serves as an important mechanism for restoring calm, balance, and well‐being, thereby contributing to positive health outcomes.^4^, ^8^, ^9^, ^10^ More important, systematic reviews highlight its role in promoting physical and mental vitality, as well as its influence on exercise adaptations, supporting recovery, optimizing performance, and contributing to long‐term health.^8^, ^11^

Rest becomes even more significant in times of chronic illness.^12^, ^13^, ^14^ Research has demonstrated that rest plays a critical role in coping strategies for managing fatigue and optimizing activity in rehabilitation programs aimed at achieving daily functional goals.^15^, ^16^ For example, activity pacing is defined as a self‐management and self‐regulatory approach aimed at the effective distribution of energy over time.^16^, ^17^ This method entails balancing periods of activity and rest to optimize energy expenditure, thereby alleviating the negative impact of fatigue on daily functioning and enhancing an active lifestyle.^16^, ^18^ Rest is a key component of activity pacing, which helps individuals with long‐term conditions (LTCs) such as arthritis or multiple sclerosis manage their energy, reduce fatigue, promote gradual increases in activity, and optimize function without worsening symptoms.^18^, ^19^, ^20^, ^21^, ^22^

In addition, promoting an active lifestyle and activity management may help manage fatigue and prevent further health decline.^16^, ^23^ However, limited studies have explored self‐management interventions, such as activity pacing, for adults with LTCs, and the available evidence, though inconsistent, shows promising results.^18^, ^22^, ^24^, ^25^ Most studies and guidelines emphasize physical activity,^26^, ^27^, ^28^ whereas the equally important bouts of rest are less specified in rehabilitation.^6^ This has implications for rehabilitation and health care, as the way rest is currently operationalized may limit the ability to assess its potential role and to provide evidence‐based recommendations regarding its optimal quality and quantity.^6^ Furthermore, advising individuals to engage in rest without clear specifications may inadvertently encourage activity avoidance,^29^, ^30^ a behavior associated with adverse health outcomes.^31^ This is particularly concerning for adults with LTCs, where activity avoidance may lead to further physical decline^17^ and impede rehabilitation progress.

Moreover, a prior review^32^ identified studies on the cultural meanings and practices of rest. However, that review was limited by an unclear methodology, including the absence of inclusion and exclusion criteria, which resulted in a small number of rest‐related studies. Furthermore, because the review was conducted nearly a decade ago, its findings may not reflect more recent developments. Recent studies have increasingly emphasized the importance of understanding rest within the broader framework of fatigue management.^14^, ^19^ For instance, a narrative review has highlighted rest as a crucial element within the multidimensional activity pacing model in rehabilitation.^19^ Relatedly, a qualitative study involving 15 adults in the United Kingdom with chronic conditions and symptoms of chronic fatigue found that promoting rest and cultivating a supportive social environment could be key elements in the rehabilitation process to enhance the effectiveness of fatigue management.^14^ These findings, coupled with the insufficient description of rest in rehabilitation and daily life, highlight the need for further research to enhance our understanding on how rest is operationalized and implemented in rehabilitation, health care, and daily life.

It is important to recognize that when the concept of rest is inadequately explained, patients may be uncertain about whether they have paced their activities appropriately.^7^, ^33^ Consequently, understanding how rest is operationalized in daily life could help guide the design of interventions incorporating rest such as activity pacing to optimize rehabilitation and health care outcomes.^34^ One effective approach to advancing the understanding of the concept of rest is to systematically examine how it has been measured and operationalized within existing literature for adults with and without LTCs. This examination may potentially reveal patterns in how rest is defined and evaluated across different studies, which, in turn, could inform the development of standardized measures for clear and actionable guidance for its implementation in rehabilitation, health care, and daily life. However, to the best of our knowledge, no comprehensive review has been conducted on the operationalization and available measurement tools for rest in the context of rehabilitation, health care, and daily life.

Therefore, we conducted a systematic scoping review to investigate the operationalization and measurement of rest among adults with and without LTCs. The primary objectives were to examine existing literature on rest within rehabilitation, health care, and daily life to assess its operationalization and implementation. Furthermore, the study sought to identify and evaluate the tools utilized for measuring rest in rehabilitative settings. This investigation aims to provide foundational insights that may guide future research and enhance the integration of rest into rehabilitative practices and daily life.

## METHODS

### Best practices and eligibility criteria

We followed the scoping review methodological framework developed and proposed by Joanna Briggs Institute^35^ and reported in accordance with the Preferred Reporting Items for Systematic Reviews and Meta‐Analyses extension for Scoping Review (PRISMA‐ScR) checklist.^36^ The eligibility criteria followed the Population, Concept, and Context model.^35^

### Population/participants

The review focused on adults with LTCs defined as conditions requiring prolonged and ongoing management (eg, stroke, arthritis).^37^ We also included adults without LTCs, such as health care professionals (HCPs), professional and nonprofessional caregivers or a combination thereof. Furthermore, adults without LTCs who were neither HCPs nor caregivers for people with LTCs were also included to ensure the review captures the full scope of relevant evidence. The inclusion of HCPs was informed by the social learning theory,^38^ which asserts that when an individual sees a model whom they identify with or admire, the model's behaviors may serve as a cue for the individual to initiate similar behaviors.^38^ Additionally, an HCP may be less willing to attempt to promote healthy lifestyles if they do not have a healthy lifestyle themselves.^39^ Therefore, an HCP's understanding and perception of rest may drive their resting behavior and advice in the context of health care and rehabilitation. Additionally, their resting practices and advice may be modeled and imitated by their patients. Moreover, caregivers are crucial to the global health care system and provide emotional, physical, and practical support, assisting with daily activities and medical care.^40^ Moreover, they are believed to deliver up to 90% of care for individuals with LTCs,^40^ and therefore their understanding of rest may also affect their resting behaviors and the advice they give in health care and rehabilitation contexts. Furthermore, adults without LTCs who were neither HCPs nor caregivers for people with LTCs were also included because rest is a universal concept relevant to both clinical and nonclinical populations. Understanding how rest is operationalized and conceptualized across diverse groups may provide valuable insights into its implementation and importance in various contexts.

### Concept

The principal concept of interest was rest. It is important to acknowledge that, although sleep and rest are closely related concepts,^34^ they differ significantly in their mechanisms.^32^ Consequently, for the purposes of this review, we prioritized understanding wakeful rest.^1^ Thus, sleep studies were excluded. Additionally, the review included findings derived from global studies seeking to understand the nature, experiences, and scope of rest in rehabilitation and health care. Moreover, the measurement tools developed purposely for adults with LTCs were included. However, studies specifically examining bed rest and rest in the concussion population were excluded. This is because the concept of bed rest and rest in patients with concussion has been extensively studied.^41^, ^42^, ^43^, ^44^ Finally, studies on rest in sports were excluded.

### Context

The scoping review included studies from global rehabilitation and health care context, with studies from across the globe and various geographical locations considered and mapped. Rest differs from person to person and is largely influenced by culture and beliefs.^45^ Therefore, synthesizing rest data worldwide may provide a holistic perspective and understanding of rest in the context of health care and rehabilitation.

### Sources of evidence

The review aimed to include a variety of studies that explored the concept of rest, including qualitative, quantitative, reviews, and concept analyses. However, we excluded nonretrieved full‐text papers, studies on resting‐state EEG, and studies into rest in domains beyond the scope of health and rehabilitation.

### Information source and search

We conducted searches in PubMed, the Cumulative Index to Nursing and Allied Health Literature (CINAHL), and Psych databases to explore the concept of rest in rehabilitation and more broadly. The last search was conducted in September 2024. Keywords used included “rest”[MeSH Terms], ‘rest advice’, “perception”[MeSH Terms], “understand”[All Fields], “experience”[All Fields], “chronic pain”[MeSH Terms], “chronic fatigue”[All Fields] as well as terms related to measurement properties example “psychometric*”[All Fields], “Reliability”[All Fields], “Validation”[All Fields] to examine the conceptualization and measurement of rest in rehabilitation contexts. These terms were combined using the Boolean operators “AND” and “OR.”^46^ The search strategy was comprehensive and not restricted to any specific LTC or non‐LTC. However, the search was restricted to studies published between 1990 and 2024, focusing on adult populations, to scope the literature from the late 20th century to the present. Finally, the bibliographies and reference lists of the identified articles were reviewed for relevant papers. See Supplementary File for the search strategies for each database.

### Study screening and selection

The search output from the databases was exported into Mendeley citation manager where duplicate articles were removed. Next, M.A. and H.B. independently reviewed the titles, abstracts, and screened full text to select potential studies for the review. Any divergent views were double checked and resolved through discussion with another coauthor (U.A.).

### Data extraction, charting process, and synthesis

Data extraction and charting were performed by M.A. Extracted information included the first author's name and year of publication, aim of the study, country, study design, and participants. The selection procedure was summarized using the PRISMA flow chart.^47^ The findings were analyzed in line with the research objectives. An inductive content analysis was conducted to synthesize qualitative and quantitative evidence and categorize descriptions^48^, ^49^ of rest in the literature. Descriptions and phrases related to rest from the selected studies were coded and subsequently categorized to identify how rest was operationalized, along with the perceived important and goals of rest. The frequency of studies related to each category was also reported.^49^ Additionally, for the second objective, which focused on the measurement of rest, the findings were reported descriptively and presented with a narrative explanation.

## RESULTS

### Screening and study characteristics of the included studies

**FIGURE 1:**
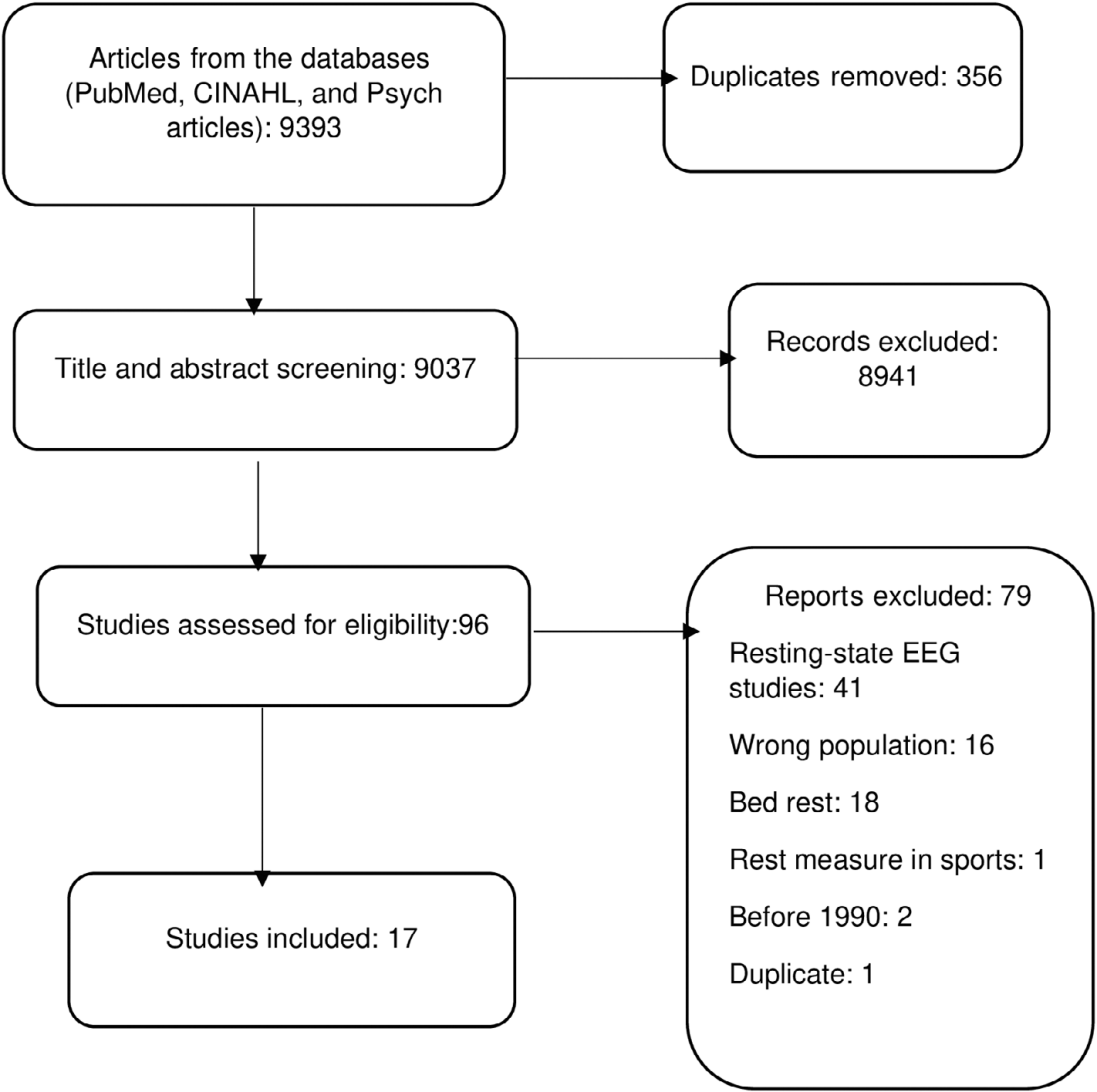
Preferred Reporting Items for Systematic Reviews and Meta‐Analyses flow chart for study screening. CINAHL, Cumulative Index to Nursing and Allied Health Literature.

Figure 1 depicts the process of article searching and screening. A total of 17 articles were included in this study.^2^, ^3^, ^7^, ^30^, ^34^, ^50^, ^51^, ^52^, ^53^, ^54^, ^55^, ^56^, ^57^, ^58^, ^59^, ^60^, ^61^ Five studies were conducted in the United States,^7^, ^34^, ^51^, ^55^, ^61^ three studies in Spain,^54^, ^58^, ^59^ two studies each in Canada^3^, ^53^ and the Netherlands.^30^, ^56^ One study each in Sweden,^57^ Denmark,^50^ Israel,^2^ and Australia.^52^ Additionally, the studies were categorized by their methodological approaches. Ten studies employed qualitative methodologies,^2^, ^3^, ^7^, ^34^, ^51^, ^52^, ^57^, ^58^, ^59^ and seven used quantitative methods.^30^, ^50^, ^53^, ^54^, ^56^, ^60^, ^61^ Moreover, eight studies involved adults with LTCs,^3^, ^30^, ^50^, ^52^, ^53^, ^54^, ^56^, ^61^ five involved mixed populations (eg, both adults with and without LTCs),^2^, ^7^, ^34^, ^57^, ^60^ three involved both professional and nonprofessional caregivers,^55^, ^58^, ^59^ and one study involved health professionals^51^ Furthermore, the review identified two measures of rest that were used in studies involving adults with LTCs.^53^, ^61^ The results are presented in Table 1.

**TABLE 1:** Characteristics of the included studies.

Author and year		Aim of the study	Country	Study design	Participants
1.	Asp, 2015,^57^	To examine the concept of rest	Sweden	Qualitative study (descriptive phenomenology)	63 participants, both people with and without LTCs
2.	Bernhofer, 2016,^7^	To report an analysis of the concept of rest	United States	Qualitative (concept analysis)	People with and without LTCs
3.	Cuesta‐Benjumea, 2011,^58^	To uncover how women caregivers of relatives with advanced dementia rest from caregiving	Spain	Qualitative study (constructivist grounded theory)	23 primary caregivers of patient with advanced dementia
4.	Cuesta‐Benjumea, 2014,^59^	To contrast family caregivers and migrant caregivers' strategies for relief from their caring role	Spain	Qualitative study (grounded theory)	40 family caregivers and caregivers for persons with dementia
5.	Evers et al., 1998,^30^	To examine the coping styles and social support impact on rheumatoid arthritis functional status	The Netherlands	Quantitative (prospective)	91 persons with rheumatoid arthritis
6.	Gibbs et al., 2011,^3^	To explore the experience of rest for women with hip and knee osteoarthritis	Canada	Qualitative study (hermeneutic phenomenology	11 women with osteoarthritis
7.	Hammond et al., 2016,^60^	To investigate how people understand rest	Global	Quantitative (survey)	More than 18, 000 people from 134 countries (people with and without LTCs)
8.	Helvig et al., 2016,^34^	To report an analysis of the concept of rest.	United States	Qualitative (concept analysis)	People with and without LTCs
9.	Jensen et al., 1995,^61^	To develop and validate a measure of strategies used by patients to cope with pain	United States	Quantitative (questionnaire development and survey)	176 chronic pain patients
10.	Jensen et al., 2012,^50^	To compare the current ‘state‐of‐the art’ treatment approach (exercise and encouragement to keep active) with a new approach (load reduction and daily rest	Denmark	Quantitative (randomized controlled trial)	100 patients with low back pain
11.	Mornhinweg et al., 1996,^51^	To describe the concept of rest from a variety of perspectives and lay the foundation for further analysis	United States	Qualitative study	10 health professionals
12.	Nurit et al., 2003,^2^	To explore the phenomenon of rest as reported in the literature and through a qualitative study	Israel	Qualitative study (grounded theory)	Rest across various health domains and exposure in the life of seven adults without LTCs
13.	Purcell et al., 2020,^52^	To explore stroke survivors' engagement in “occupations” during stroke rehabilitation	Australia	Qualitative study (descriptive phenomenology)	8 survivors of stroke
14.	Racine et al., 2024,^53^	To describe the development of Activity Management Inventory for Pain	Canada	Quantitative (questionnaire development and survey)	332 patients with chronic pain symptoms
15.	Rodero et al., 2011,^54^	Relationship between behavioral coping strategies and acceptance in patients with fibromyalgia syndrome	Spain	Quantitative	167 patients with fibromyalgia syndrome
16.	Rush et al., 2024,^55^	To explore how professional caregivers make sense of and practice, and meaning of rest	United States	Qualitative	25 professional caregivers
17.	Steultjens et al., 2001,^56^	To examine coping styles as predictors of pain and disability in osteoarthritis	The Netherlands	Quantitative	71 persons with hip and knee osteoarthritis

Abbreviation: LTC, long‐term condition.

### Operationalization of concepts of rest

#### Physical rest: Involves stopping or partaking in low energy activities

**FIGURE 2:**
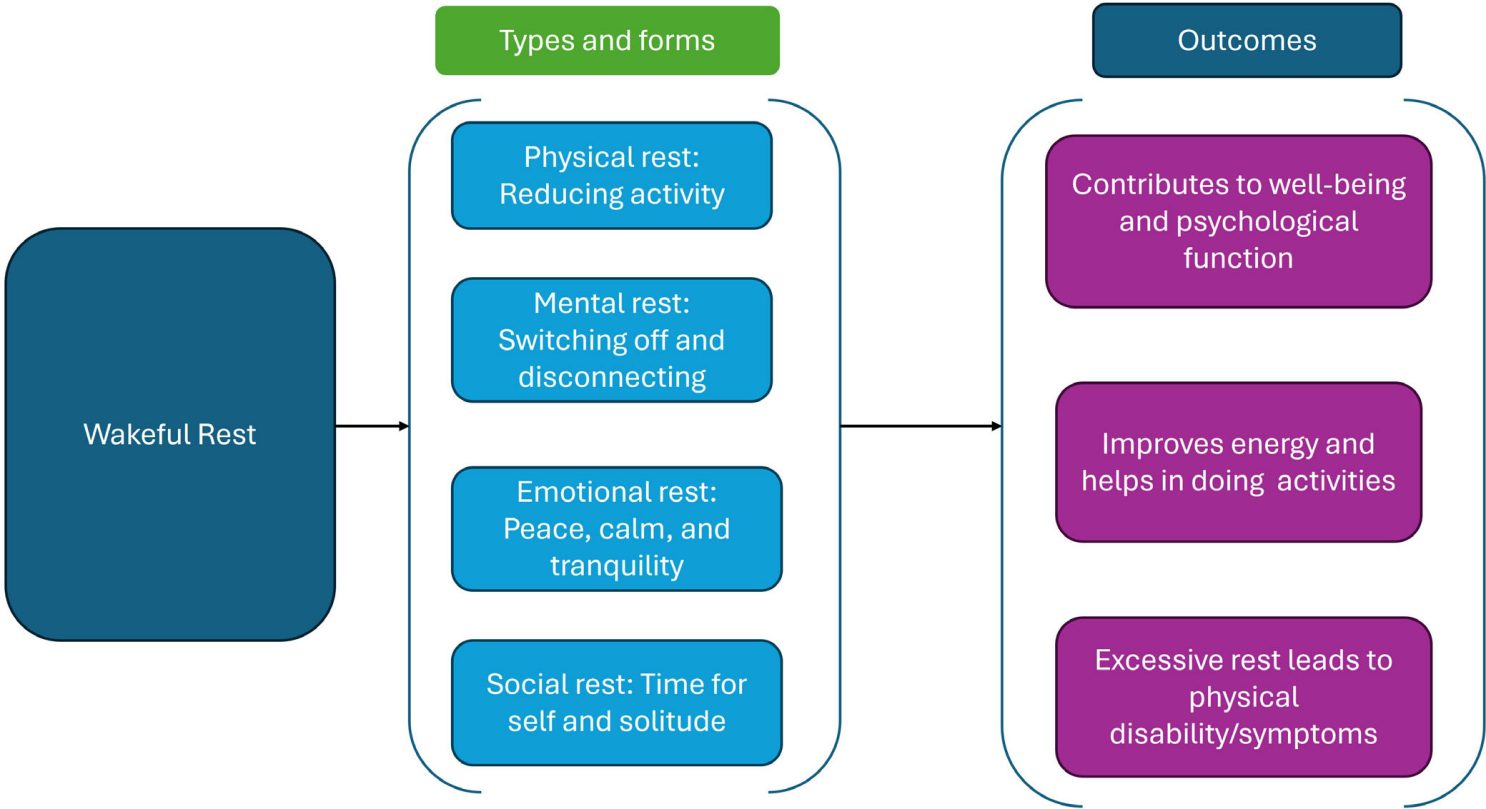
Identified types and forms of wakeful rest and its importance among adults with and without long‐term conditions.

Across nine studies,^7^, ^30^, ^34^, ^50^, ^51^, ^53^, ^54^, ^56^, ^61^ the concept of rest was operationalized as a reduction, cessation, or limitation of activity (Figure 2). Of these, six studies focused on adults living with LTCs,^30^, ^50^, ^53^, ^54^, ^56^, ^61^ two studies involved mixed populations comprising both adults with and without LTCs,^1^, ^2^ and one study was conducted with nurses.^51^ Helvig et al.^34^ examined the concept of rest within the health care context, asserting that rest involves the deliberate cessation of activity to promote both physical and mental health. In a comparable vein, rest has been defined as a fundamental human need that is intentional, temporary, and restorative and involves a purposeful cessation, reduction in physical, mental, or spiritual activities.^7^ Similarly, in a randomized controlled trial involving 100 adults with low back pain, rest was operationalized through specific guidelines. Participants were instructed to avoid hard physical activity and to include two 1‐hour rest periods in their daily routines, during which they were encouraged to lie down to relieve back strain and promote recovery.^50^

#### Mental rest: Involves detachment and disconnecting

Four studies^2^, ^57^, ^58^, ^59^ reported that rest could involve detachment and switching off physically and mentally from routine responsibilities and obligations (Figure 2). Among these, >75% of the studies were conducted with adults without LTCs.^2^, ^58^, ^59^ In a qualitative study involving 40 nonprofessional caregivers, rest was defined as a disconnection from the caregiving identity, and some forms of caregivers' rest were characterized as “false exits” from this identity.^59^ Similarly, in a qualitative study of mixed populations, rest was defined as adhering to a rest rhythm, which entails taking breaks throughout the day (eg, disengaging from routine tasks) and temporarily redirecting one's attention from nonrestful activities to restful ones.^57^

*“*When mentally demanding activities have been performed for a while and the experiences of non‐rest are evident, there is a need to switch on to a mentally restful activity, which could be a physical activity.*”* ^57^

#### Emotional rest: Involves experience of peace, joy, and tranquility

Five studies reported that rest is associated with key qualities, specifically peace, joy, comfort, calmness, and inner tranquility.^34^, ^52^, ^55^, ^57^, ^60^ (Figure 2). Three studies included mixed populations,^34^, ^57^, ^60^ one focused on professional caregivers,^55^ and one involved adults with LTCs.^52^ In a study involving a sample of eight survivors of stroke exploring occupational engagement in rehabilitation, participants described rest as a key element in their recovery process, emphasizing the importance of savoring and valuing quiet moments.^52^ These moments of rest were considered an essential component of their overall rehabilitation experience. Participants noted that rest provided an opportunity for mental and physical recuperation, allowing them to recharge from the demands of therapy and other rehabilitation activities.^52^ Additionally, in a sample of 25 professional caregivers, participants described their definition of rest as a strategic defense mechanism, essential for self‐restoration and the caregivers characterized rest as an indispensable source of joy in their daily lives.^55^ Other studies have described rest as a pathway to calmness and tranquility.^34^, ^57^, ^60^

#### Social rest: Time for self and solitude

Two studies operationalized rest as time for self‐care and personal engagement^2^, ^52^ (Figure 2). One study involved a mixed population,^2^ and the other focused on adults with LTCs.^52^ Purcel and colleagues^52^ found that rest was perceived as a time for self‐reflection and personal engagement. Although rest provided opportunities for social interaction with other individuals on the ward, it was also regarded as “time to myself,” allowing participants to engage in meaningful occupations such as crocheting and reading. Furthermore, rest facilitated reflection on their prestroke lifestyle, contributing to a sense of continuity and personal identity. In a qualitative study involving a mixed population within a health care setting, participants described true rest as a personal experience, often captured by the phrase “between me and myself.”^2^

### The perceived importance and goals of rest

#### Rest contributes to well‐being and psychological function

Four studies^3^, ^7^, ^34^, ^60^ indicated that rest may contribute to both well‐being and psychological functioning. (Figure 2).

Gibbs and Klinger^3^ examined the experience of rest in 11 women with osteoarthritis aged 60 to 75 years and reported that participants believed rest helped them to minimize physical damage and continue engaging in valued activities. Additionally, participants reported that rest was important for their mental and emotional well‐being, as it enabled them to remain active in their relationships and prevented osteoarthritis from overwhelming their lives.^3^

This observation was further substantiated by a comprehensive global survey encompassing a diverse population, including both adults with and without LTCs.^60^ The survey, which included over 18,000 participants from 134 countries, revealed significant findings regarding the relationship between rest and subjective well‐being. Individuals who reported greater amounts of rest in the preceding 24 hours exhibited higher scores in subjective well‐being. In contrast, participants who felt a need for more rest and perceived that they had received less rest compared to others demonstrated lower scores in subjective well‐being.^60^ Other studies have highlighted the benefits of adequate rest in enhancing mental clarity and reducing worry and perceptions of danger.^7^, ^34^

#### Improve energy and help in doing activities

Four studies^2^, ^7^, ^34^, ^51^ reported that rest helped to improve energy and performance of daily activities. (Figure 2).

In a sample of seven adults without LTCs, Nurit and Michall found that rest was appraised as a positive occupation.^2^ Participants reported that rest facilitated daily performance by providing a necessary break from routine activities. Furthermore, the individuals in the Nurit and Michall^2^ study indicated that rest played a crucial role in restoring their energy, enabling them to effectively manage their daily tasks and responsibilities. As one participant noted, “Rest gives me the power to continue,” and another stated, “After a rest, I am refreshed and able to continue with my daily activities, such as household tasks.”^2^ In the same vein, a qualitative study involving a sample of 10 nurses reported that rest was perceived as essential for recharging not only physical energy but also mental and spiritual energies.^51^

Two conceptual studies reported on rest enhancing energy level.^7^, ^34^ Helvig and colleagues,^34^ in their conceptual analysis of rest, underscored that rest is a fundamental human need, vital for maintaining physical and psychological well‐being. They argued that rest serves as a crucial mechanism for replenishing energy levels and fostering the body's natural recovery processes. According to their analysis, rest not only supports improved physical energy but also plays a significant role in restoring health by allowing the body to heal from various conditions. Similarly, Bernhofer's (2016)^7^ conceptual analysis of rest linked it to enhanced mental and cognitive energy and the relief of cognitive and emotional stress. Rest was also identified as a key factor in promoting life balance and overall well‐being.^7^

#### Relationship between rest and physical disability/symptoms

Four studies^30^, ^50^, ^54^, ^56^ examined the relationship between rest and physical disability or physical symptoms (Figure 2). Of these, three studies identified an association between rest and increased physical disability. A study of 71 patients with hip osteoarthritis and 119 patients with knee osteoarthritis demonstrated that a passive coping strategy involving excessive rest was linked to greater disability in patients with knee osteoarthritis, though no such association was found in those with hip osteoarthritis.^56^ Additionally, a study of 91 individuals with rheumatoid arthritis revealed that frequent reliance on passive pain‐coping strategies, such as excessive worrying and resting, predicted a decline in functional status after 1 year.^30^ On the other hand, a randomized controlled trial examined the comparative effects of rest versus exercise in adults with low back pain found that a rest regimen, when combined with an activity‐based approach, yielded similar benefits to those of maintaining regular physical activity in terms of pain management.^50^

#### Measurement of rest in adults with long term conditions with fatigue/pain symptoms

The scoping review identified two measures used to assess rest in adults with LTCs: the Chronic Pain Coping Inventory (CPCI)^61^ and the Activity Management Inventory‐Pain (AMI‐P).^53^

The CPCI^61^ items were developed by researchers specializing in chronic pain and have been validated for use in adults with chronic pain, as well as their significant others. The CPCI evaluates coping strategies across nine subscales, categorized into two domains: “illness‐focused” strategies (eg, guarding, resting, seeking assistance) and “wellness‐focused” strategies (eg, exercise/stretching, relaxation, task persistence, coping self‐statements, pacing, and seeking social support). The rest‐related items specifically assess resting behaviors such as resting as much as possible, sitting in a chair or recliner, lying down in bed, retreating to a room to rest, and lying on a sofa.

Similarly, the AMI‐P^53^ items were developed by an international team of researchers and experts specializing in chronic pain and activity management. These items have been validated in adults with chronic pain and are structured around three key factorial components: rest, alternating activity, and planned activity. The rest component comprises items that assess rest‐related behaviors, including lying down, sitting, limiting engagement to essential activities, and taking rest breaks as needed. The participants were instructed to report the frequency, in days, of their use of rest as a strategy for managing activity.

The existing scales primarily assess aspects of rest activity or behaviors associated with rest, emphasizing how individuals engage in rest practices. However, these instruments appear to lack input from patients during their development.

## DISCUSSION

The review aimed to explore the operationalization and measurement of rest in rehabilitation. The findings reveal that rest is defined by key elements: cessation or engagement in low‐energy activities (eg, physical rest); detachment and disconnecting (eg, mental rest); experience of peace, joy, and tranquility (emotional rest); and time for self and solitude (eg, social rest). The operationalization of rest highlighted both divergence and convergence between adults with and without LTCs. Although all groups conceptualized rest in terms of physical, emotional, and social aspects, mental rest was predominantly emphasized by adults without LTCs. The effects and perceived goal of rest were identified as improvement to overall well‐being, psychological functioning, improved energy levels, and support in performing daily activities. However, “excessive” rest was associated with the opposite effects; increased physical symptoms and disability. Lastly, comprehensive measurement tools for evaluating rest in adults with LTCs were notably scarce.

Several studies operationalized rest as involving stopping/reduction of activity, where minimal physical and cognitive energy is expended, to promote both physical and mental health. These findings suggest that rest is not only essential for recovery from acute injuries and postsurgical interventions but also serves as a preventative measure, supporting well‐being by providing opportunities for mental and physical restoration. In rehabilitation, structured rest periods are often prescribed to prevent overexertion and to ensure that patients can restore energy and achieve optimal functioning in daily living.^22^ Additionally, strategically slowing down is recognized as a preventative measure and an essential factor in pacing activity,^22^, ^24^, ^62^, ^63^ optimizing function, and promoting an active lifestyle in rehabilitation.^16^, ^22^ Our review also identified that rest is frequently conceptualized as a detachment or disconnection from core activities. This operationalization includes switching off; a temporary shift in focus from active, task‐oriented pursuits to restorative activities. The process of detachment is strongly associated with improved psychological functioning and enhanced motivation to engage in activities.^64^ Furthermore, findings from five studies identified key qualities associated with rest, specifically peace, joy, comfort, calmness, and inner tranquility. In addition, some studies emphasize that rest is a state of savoring and valuing quiet moments. This highlights that the experience of rest produces positive emotional affectivity and may be associated with well‐being and self‐care. Moreover, the emphasis on time for self and solitude in the process of resting may highlight the importance of personal space and self‐reflection in rehabilitation. Together, the conceptualization of rest identified in the present review can be broadly classified into four distinct categories: physical rest, mental rest, emotional rest, and social rest. These categories highlight the multidimensional nature of rest and its importance in fostering holistic well‐being.

The conceptualization of rest reveals both similarities and differences between adults with and without LTCs. Across the studies, there was a clear convergence in how rest is conceptualized, with the physical, emotional, and social dimensions consistently identified as key components of rest for both groups. This reflects a broader understanding of rest that goes beyond mere physical to include emotional and social elements that are essential for overall well‐being and self‐care. Such a comprehensive approach highlights the multidimensional nature of rest, underscoring its importance in promoting holistic health. However, divergence arises in the emphasis placed on mental rest. This was predominantly operationalized by adults without LTCs, who tended to focus on mental detachment as a vital dimension of rest. This prioritization of mental rest could be attributed to the fact that adults without LTCs may have the capacity to devote more attention to psychological recovery,^1^, ^64^ as they are less burdened by the ongoing demands of managing a chronic physical condition. In contrast, adults with LTCs may prioritize physical, social, emotional forms of rest as more immediately necessary, given the daily management of their conditions and the associated physical or emotional burdens. These findings suggest that although the core understanding of rest remains similar across populations, the operationalization and prioritization of mental rest may differ. This highlights the importance of tailoring rest interventions to the specific needs of individuals, particularly in the context of physical LTCs, where physical, social, and emotional rest may take precedence. Future research should explore how various health conditions influence the prioritization of different forms of rest and examine how interventions can be better aligned with these needs.

Importantly, the operationalization of rest and perceived goals appears to contribute to well‐being, improve energy, and enhance the importance of daily performance in activities^3^, ^7^, ^34^, ^60^ and could be a positive occupation in the context of self‐care, leisure, and activity of daily living.^2^, ^3^ Consequently, when rest is recognized as a valuable, purposeful activity, individuals are better able to manage their energy and achieve rehabilitation goals, improving their overall functioning and well‐being. Nevertheless, there appears to be an inverted U‐shaped relationship between rest and health outcomes, indicating that both insufficient and excessive rest can have deleterious effects on physical and mental health. Too little rest may lead to burnout, fatigue, and increased stress, and too much rest might contribute to decreased performance or even depression.^15^, ^60^, ^65^, ^66^ It is also important to consider rest not as the opposite of activity, nor merely as a coping strategy. Rest is viewed negatively, particularly in the coping literature, where it is frequently conflated with avoidant coping.^17^, ^56^ This negative perception of rest may exacerbate physical disability and symptoms in adults with LTCs.^17^, ^30^, ^54^, ^56^ The existing association between rest and increased physical disability and symptoms may be attributed to rest being used as a reactive measure to reduce activity and mitigate symptoms, rather than as a proactive restorative approach in rehabilitation. Additionally, insights from the activity pacing literature demonstrate that rest and activity are not opposites, but rather mutually reinforcing variables.^16^, ^22^, ^25^, ^55^, ^67^ When balanced appropriately, rest may support activity by providing periods of restoration and recovery, making it easier to perform daily activities.^55^ Consequently, by understanding rest as a proactive restorative process and positive experience rather than an avoidant or passive behavior, we can better appreciate its contribution to sustaining a balanced and healthy lifestyle.

We identified two measures for rest in adults with LTCs.^53^, ^61^ Although they offer important insights into coping strategies and activity management within the context of chronic pain, the current resting scales appear to have some limitations. The existing rest scales were developed and tailored specifically for chronic pain patients' behaviors and not adults with LTCs and symptoms of fatigue. Consequently, there exists a notable absence of a resting measure tailored to adults with LTCs with symptoms of fatigue. Moreover, the current content of rest scales may be limited due to the absence of patient input during their development.^68^ Again, the present construct of rest seems to prioritize and frame it to be maladaptive strategies rather than rehabilitative ones.^68^ Therefore, the prevailing conceptualization of rest may be influenced by a fear‐avoidance model.^69^, ^70^ This perspective has the potential to contribute to adverse health outcomes, such as increased fatigue and pain, and physical disability.^30^, ^56^ Collectively, the shortcomings of existing measures of rest, coupled with their limitations in the context of rehabilitation, highlight a clear need for further examination in future research. Importantly, existing measures of rest may not reflect its operationalization within the context of fatigue management, highlighting the need for further exploration in this population.

Taken together, the findings of this study may carry important implications for the design of activity pacing interventions. Historically, these interventions have primarily focused on advising patients to rest to facilitate increased activity levels.^22^, ^24^ However, the current findings suggest that individuals may conceptualize and operationalize rest differently, which could result in different health outcomes. This variability in the understanding and operationalization of rest could explain the inconsistent evidence in pacing literature.^22^, ^24^, ^71^ Additionally, it is important to highlight that we should not only focus on the quality and distribution of physical activity but also on the quality and distribution of rest. Consequently, we propose that balancing physical activity with rest, and optimizing the quality of rest, might be crucial elements in the success of activity pacing interventions. Furthermore, among the measures of rest identified, the CPCI conceptualizes rest as illness focused, whereas relaxation is viewed as promoting general well‐being. However, some studies treat these concepts as interchangeable.^2^, ^3^ This divergence may indicate that existing measures often associate rest with avoidance,^17^, ^56^ implying that rest is seen as a passive response to illness/symptoms, which could reinforce a state of dependency. In contrast, relaxation may be viewed as a proactive psychological process that enhances emotional and physical health. Given the limited measures and underdeveloped understanding of rest,^7^ more studies are needed to explore whether rest and relaxation are synonymous or distinct concepts. Such exploration could improve health interventions and help develop tools that support both physical restoration and mental well‐being.

## LIMITATIONS AND FUTURE DIRECTIONS

An important limitation of the present review was that, in studies involving mixed populations, it was difficult to fully distinguish the operationalization of rest between adults with and without LTCs. This lack of separation makes it challenging to assess the unique perspectives and needs of each group, which may have implications for interpreting the findings. Future research could aim to explore these distinctions more clearly, potentially enhancing the understanding of rest in different populations while building on the current study's contributions.

Additionally, a pronounced geographical bias was evident in the studies, with the majority concentrated in developed countries. Specifically, >95% of the research was conducted in high‐income regions, potentially limiting the applicability of the findings to non‐Western and low‐ and middle‐income countries. Even within high‐income nations, certain countries were significantly underrepresented. Consequently, there is a clear need for more globally inclusive research on rest, particularly in diverse geographical and socioeconomic contexts.

Moreover, the limited availability of validated rest measures for adults with LTCs with fatigue highlights a gap in assessing rest within this population. Existing measurement tools may not adequately capture the specific rest needs of this population, such as the quality of rest. Alternatively, the development of a novel rest measure with input from patients and HCPs may be necessary for developing tailored interventions.

Despite these limitations, it is important to acknowledge that this scoping review adhered to internationally recognized standards^35^, ^36^ and represents the first effort to explore the operationalization and measurement of rest in rehabilitation and health care. Consequently, the identified gaps may serve as valuable guidance for future research.

## CONCLUSION

The study provides important insights into the operationalization and measurement of rest in the daily lives of adults with and without LTCs. The findings reveal that rest is operationalized through several key components, including the cessation of activities, engagement in low‐energy tasks, detachment from daily stressors, and the experience of peace, joy, and tranquility. Rest is also associated with moments of self‐reflection and solitude. The operationalization of rest demonstrates both convergence and divergence between adults with and without LTCs. Although all groups defined rest in terms of physical, emotional, and social aspects, mental rest was primarily emphasized by adults without LTCs. The study suggests that rest can contribute to improved energy levels, enhanced well‐being, and a greater capacity to engage in daily activities. However, it also highlights that “excessive” rest may lead to negative outcomes, such as the worsening of physical symptoms and increased disability. Furthermore, the review identifies a significant gap in the availability of comprehensive measurement tools to assess rest, particularly in adults with LTCs. This gap highlights the urgent need for the development of novel, validated instruments capable of more effectively measuring rest. Such tools could not only advance our understanding of the role of rest in daily life, health, and rehabilitation, but also inform more personalized and evidence‐based approaches in both clinical practice and research. Furthermore, we anticipate that validated measures of rest will support the systematic identification of rest‐related needs among people with LTCs and their caregivers, thereby contributing to more holistic and person‐centered care.

## DISCLOSURES

None.

## Supporting information


**Data S1.** Supporting Information.
