# Supplementary material for: How is wakeful rest operationalized and measured in daily life among adults with and without long‐term conditions? A systematic scoping review
Source: PM R. 2025 Sep 4;18(3):332–44. doi: 10.1002/pmrj.70008 (PMC13001139; doi:10.1002/pmrj.70008)
Supplement: Supplementary file 1 — Data S1. Supporting Information. [file PMRJ-18-332-s001.docx]

**PubMed**

(("rest"[MeSH Terms] OR "rest"[All Fields]) OR rest advice AND ("comprehension"[MeSH Terms] OR "comprehension"[All Fields] OR "understand"[All Fields] OR "understanding"[All Fields] OR "understands"[All Fields] OR "understandability"[All Fields] OR "understandable"[All Fields] OR "understandably"[All Fields] OR "understandings"[All Fields] OR "perception"[MeSH Terms] OR "perception"[All Fields] OR ("experience"[All Fields] OR "experiences"[All Fields]) OR "attitude*"[All Fields] OR (("coped"[All Fields] OR "copes"[All Fields] OR "coping skills"[MeSH Terms] OR (("coping"[All Fields] AND "coping skills"[All Fields]) OR "coping"[All Fields] OR "copings"[All Fields]) OR (("develop"[All Fields] OR "developing"[All Fields] OR "developments"[All Fields] OR "develops"[All Fields] OR "development"[All Fields] OR "psychometric*"[All Fields] OR "Reliability"[All Fields] OR "internal consistency"[All Fields] OR "intraclass correlation*"[All Fields] OR "ICC"[All Fields] OR "Validity"[All Fields] OR "Validation"[All Fields]) AND ("manage"[All Fields] OR "managed"[All Fields] OR "management s"[All Fields] OR "managements"[All Fields] OR "managing"[All Fields] OR "management"[All Fields] OR "disease management"[MeSH Terms] OR ("disease"[All Fields] AND "management"[All Fields]) OR "disease management"[All Fields])) OR ("chronic pain"[MeSH Terms] OR ("fatigue"[All Fields] OR "chronic fatigue"[All Fields] OR ("chronic"[All Fields] AND "pain"[All Fields]) OR "chronic pain"[All Fields])) AND ("inventoried"[All Fields] OR "inventory s"[All Fields] OR "inventories"[All Fields] OR "inventory"[All Fields]))) AND ("adult"[MeSH Terms] AND 1990/01/01:2024/12/31[Date - Publication])) NOT ("sleep"[MeSH Terms] OR "sleep"[All Fields] OR "sleeping"[All Fields] OR "sleeps"[All Fields] OR "sleep s"[All Fields])

**CINAL Ultimate**

**S1**: TI (rest)

**S2**: TI Sleep

**S3**: TI (S1 NOT S2)

**S4**: TI (advice OR operationalization OR implementation OR Understanding OR Perception OR perceived OR practice OR experience OR attitude)

**S5**: TX (Development OR "psychometric*" OR "Reliability" OR "internal consistency" OR "intraclass correlation*" OR "ICC" OR "Validity" OR "Validation" OR "responsiveness" OR ‘Activity management OR ‘chronic pain inventory’)

**S6**: TI (S4 OR S5)

**S7**: TI (S3 AND S6), **Narrow by SubjectAge:**- all adult

**PsycArticles via ProQuest)**

abstract(rest) AND abstract (advice OR operationalization OR implementation OR Understanding OR Perception OR perceived OR practice OR experience OR attitude) AND abstract(Development OR "psychometric*" OR "Reliability" OR "internal consistency" OR "intraclass correlation*" OR "ICC" OR "Validity" OR "Validation" OR "responsiveness" OR ‘Activity management OR ‘chronic pain inventory’ OR fatigue OR chronic fatigue)
